# Sociodemographic determinants of knowledge and attitude in the primary prevention of cervical cancer among University Tunku Abdul Rahman (UTAR) students in Malaysia: preliminary study of HPV vaccination

**DOI:** 10.1186/s12889-019-7764-3

**Published:** 2019-11-05

**Authors:** Maiza Tusimin, Chek Lo Yee, Nur Zarifah Syahmi Abdul Razak, Mohamad Izwan Zainol, Halimatus Sakdiah Minhat, Zulida Rejali

**Affiliations:** 1 Department of Obstetrics and Gynaecology, Faculty of Medicine and Health Sciences, Universiti Putra Malaysia, 43400 Serdang, Selangor Malaysia; 2 Department of Community Health, Faculty of Medicine and Health Sciences, Universiti Putra Malaysia, 43400 Serdang, Selangor Malaysia

**Keywords:** Human papillomavirus (HPV) vaccination, Sociodemographic, Cervical cancer

## Abstract

**Background:**

Cervical cancer is the third most common cancer affecting women around the world in which the Human Papillomavirus (HPV) is the one of the recognized causative agent affecting women health. In response to this health issue, the Malaysian government had officially implemented the HPV immunisation programme for secondary schoolchildren in 2010 at the age of 13 years old and above. The purpose of this study is to investigate the sociodemographic determinants of knowledge and attitude among students of Universiti Tunku Abdul Rahman (UTAR) towards the HPV vaccination programme.

**Methods:**

A cross-sectional study was conducted using self-administered questionnaires, recruiting 374 UTAR’s students as the respondents by using convenience sampling method. Respondents were categorized as having good/poor level of knowledge and positive/negative attitude towards HPV vaccination.

**Results:**

Over half of the respondents were females (64.5%) and the majority were aged 20 years old and below (55.8%). Generally, 54.7% of the total respondents had a high level of knowledge towards HPV vaccine while 57.5% of the total respondents showed a negative attitude towards HPV vaccine. Female respondents aged 20 years old and below showed good knowledge (56.4%) and a more positive attitude (55.8%) towards HPV vaccine. Students from the Faculty of Medicine and Health Sciences (FMHS) exhibited higher knowledge (67.3%) and positive attitude (62.4%) as compared to the Faculty of Accountancy and Management (FAM) which showed only 32.7% of knowledge and 37.6% of positive attitude towards the HPV vaccination.

**Conclusion:**

The majority of UTAR students possess good knowledge regarding HPV vaccination. Nonetheless, they demonstrated a negative attitude towards HPV vaccination, depicting the necessity to impart and further intensify the sense of health awareness among all students, especially among male students. The judicious use of social media apart from the conventional mass media should be an advantage as to enhance the practice of HPV vaccination among them and thereafter minimize the health and economic burdens of cervical cancer.

## Key messages

This research article mainly studied the sociodemographic determinants of knowledge and attitude towards HPV vaccination in the primary prevention of cervical cancer among students of Universiti Tunku Abdul Rahman (UTAR).

## Introduction

### Background

Worldwide, cervical cancer is one of the most common cancers in women [1]. In Malaysia, it is the third most common cancer affecting women and the fifth most common cancer for the entire general population [2]. Cervical cancer constitutes 8.4% of the total female cancers in this country [2]. The cervical cancer screening programme was established in Malaysia in the year 1969 as a secondary prevention measure of cervical cancer among the target group of women aged 20 to 65 years and many cancer awareness programmes have been launched over the years. Unfortunately, despite the intensive program, the number of cervical cancer had failed to reduce [3]. Therefore, the government has made a drastic measure by implementing free HPV vaccinations in 2010 for schoolchildren as a primary prevention measure.

In 2007, a National Immunisation Survey regarding HPV and HPV vaccination among adult women in the United States discovered that the awareness of HPV was 84.3% while the awareness of HPV vaccine was 78.9% [4]. There was also a study conducted in China to determine the knowledge and attitude of HPV and HPV vaccines among women living in metropolitan and rural regions of China. Only 15% of the women in that study had heard of HPV. About 84.6% of the respondents were willing to be vaccinated if the vaccines were made available for them [5].

Several studies have been carried out to determine the level of awareness of HPV vaccination. For instance, in Melaka, 77.6% of secondary schoolgirls have heard about HPV vaccination. Meanwhile, a study among female university students showed that only 10.3% had heard of the HPV vaccine. A majority of them gained knowledge by newspapers, followed by friends and other public media such as magazines, television and radio. Only 48% of the students had the intention to receive HPV vaccination while the rest refused due to their concern regarding the safety and efficacy of the new vaccine. Therefore, this study was conducted to identify and focusing on the sociodemographic determinants of knowledge and attitude among students of Universiti Tunku Abdul Rahman (UTAR) towards the HPV vaccination programme.

## Methods

### Study design

This cross-sectional study was conducted among students of Universiti Tunku Abdul Rahman (UTAR), is a private university located in the Sungai Long Campus, Selangor, Malaysia. The recruitment was carried out by distributing the questionnaire to the students. Only local undergraduate students were selected in this study. The questionnaire had been obtained from published article [6].

### Settings

This study was carried out from March to September 2013.

### Participants

This study was conducted among students of Universiti Tunku Abdul Rahman (UTAR) which in the Faculty of Accountancy and Management (FAM) and Faculty of Medicine and Health Science (FMHS). Only local undergraduate students aged 19 to 23 were selected whereas all international students were excluded in this study.

### Data collection

#### Instruments

Sociodemographic characteristics of the respondents-Several factors were identified under the sociodemographic section which included age, gender, parental income and faculties. The questionnaire was pre-tested prior to data collection. Knowledge and attitude towards HPV vaccination-A validated questionnaire was used with a Cronbach’s coefficient alpha value of 0.92. A previous study looked into the sociodemographic determinants of knowledge on HPV vaccination among Iranian women living in Malaysia. Score of ‘1’ was given for any correct answer and ‘0’ for any incorrect answer or those who answered ‘do not know’. The median score of 6.0 for knowledge was used to categories good and poor levels of knowledge. Scores higher than the median were categorized as “good knowledge” while “poor knowledge” referred to scores lower than the median. A 4-point Likert scale was used to measure the attitude score, with ‘1’ indicating strongly agree, ‘2’ for somewhat agree, ‘3’ for somewhat disagree and ‘4’ for strongly disagree. An ‘overall attitude score’ was used for the purpose of analysis which referred to the actual total score of attitude from 18 questions for each respondent, divided by the expected total maximum score of attitude and multiplied by 100. Scores between 0 to 47 were labeled as ‘negative attitude’ and 48 to 100 as ‘positive attitude’.

### Statistical analysis

All the data in this study were analyses by using the Statistical Package for the Social Sciences Version 21.0 which is predictive analytic software. Continuous data were described by mean, median and standard deviation while categorical data were described by percentage. The Chi-Square test was used to identify the association between the variable of categorical data and Kruskal-Wallis test was used for categorical data that were not normally distributed. The standard *p* value in this study was *p* = 0.05. Any p value smaller than 0.05 (*p* < 0.05) was considered significant and vice versa.

## Results

### Sociodemographic characteristics of the respondents

Table 1 shows the distribution of respondents according to their sociodemographic characteristics. Most of the respondents were 20 years old and below (55.8%). Out of the 369 students, female had the highest frequency which was 238 students (64.5%) and majority of the respondent were Chinese (91.9%). The Faculty of Accountancy and Management (FAM) had 197 students (53.4%), which contributed to the highest number of respondents compared to Faculty of Medicine and Health Science (FHMS). There was only one respondent who was married. The other 368 students (99.7%) were single. The majority of the students’ parental income was below than RM 3000 (63.1%).

**Table 1:** Sociodemographic characteristics of the respondents

Sociodemographic characteristics of the respondents		
Variables	Respondent (*N* = 369)	
	Frequency (*n*)	Percentage (%)
Age		
≤ 20 years old	206	55.8
> 20 years old	163	44.2
Gender		
Male	131	35.5
Female	238	64.5
Ethnicity		
Malay	1	0.3
Chinese	339	91.9
Indian	21	5.7
Others	8	2.2
Faculty		
FMHS	172	46.6
FAM	197	53.4
Parents’ income		
≤ RM3000	233	63.1
≥ RM 3000	136	36.9

### Respondent’s knowledge towards HPV vaccine

Table 2 shows the distribution of respondent’s knowledge towards HPV vaccination. The question that most respondents answered correctly (“Yes”) was “What is the best age to get the vaccine?” where 75.9% of them responded correctly. On the other hand, the question that most respondents answered wrongly (“No”) was “How many types of HPV vaccines are available?”. Only 14.1% of the respondent’s answered this question correctly. Out of the 12 questions, 7 questions were reported to have 50% or more respondents who managed to answer them correctly. The other five questions were not answered correctly by the majority of the respondents.

**Table 2:** Distribution of respondent’s knowledge towards HPV vaccine

Distribution of respondent’s knowledge towards HPV vaccination		
Questions	Frequency (%)	
	Yes*	No*
How many types of HPV vaccine are available?	52 (14.1)	317 (85.9)
How much is it per dose?	83 (22.5)	286 (77.5)
What is the best age to get the vaccine?	280 (75.9)	89 (24.1)
How many times women should be vaccinated against HPV?		
	249 (67.5)	120 (32.5)
Men would be benefit from HPV vaccination?	129 (35.0)	240 (65.0)
Do you think HPV vaccine is more useful for women who have multiple sexual partners?		
	207 (56.1)	162 (43.9)
Do you think women who received of HPV vaccine do not need to do regular pap smear?		
	204 (55.3)	165 (44.7)
Can HPV vaccine cause genital warts?	119 (32.2)	250 (67.8)
Can HPV vaccine cure cervical cancer?	189 (51.2)	180 (48.8)
Can HPV vaccine cure genital warts?	145 (39.3)	224 (60.7)
Can pregnant women take HPV vaccine?	198 (53.7)	171 (46.3)

*yes: correct answer, *no: wrong answer and no information

### Association between sociodemographic characteristic and knowledge

Table 3 shows the association between sociodemographic characteristic and knowledge involved in this study. The *p*-value (*p* < 0.05) showed a significant association. This study showed female students having good knowledge (127.9%) compared to male students (72.1%) towards the HPV vaccination. Besides, the knowledge of HPV vaccination in both faculties of FAM and FMNS also showed the differences. FAM contributed the higher knowledge (111.1%) compared to FMHS which is only 88.9% towards the HPV vaccination knowledge.

**Table 3:** Association between sociodemographic characteristic and knowledge towards HPV vaccination

Sociodemographic characteristic	Knowledge towards HPV vaccine				Total	*p*-value
	Poor		Good			
	*n*	%	*n*	%		
Age						
≤ 20 years old	92	55.1	114	56.4	111.5	0.796^a^
> 20 years old	75	44.9	88	43.6	83.5	
Gender						
Male	70	41.9	61	30.2	72.1	0.019^a^
Female	97	58.1	141	69.8	127.9	
Parents income						
≤ RM 3000	127	76.0	106	52.5	128.5	0.001^a^
> RM 3000	40	24.0	96	47.5	71.5	
Faculty						
FMHS	36	21.6	136	67.3	88.9	0.001^a^
FAM	131	78.4	66	32.7	111.1	

^a^Chi-square test, ^b^Kruskal Wallis test, *p-value < 0.05 show significant association

### Association between sociodemographic characteristics and attitude

Table 4 shows the association between attitude and the sociodemographic characteristics involved in this study. The *p*-value (*p* < 0.05) showed a significant association.

**Table 4:** Association between sociodemographic characteristics and attitude towards HPV Vaccination

Sociodemographic characteristic	Attitude towards HPV vaccine				Total	*p*-value
	Negative		Positive			
	*n*	%	*n*	%		
Age						
≤ 20 years old	109	51.4	97	61.8	113.2	0.047^a^
> 20 years old	103	48.6	60	38.2	86.8	
Gender						
Male	92	43.4	39	24.8	68.2	0.001^a^
Female	120	56.6	118	75.2	131.8	
Parents income						
≤ RM 3000	139	65.6	94	59.9	125.5	0.262^a^
> RM 3000	73	34.4	63	40.1	74.5	
Faculty						
FMHS	74	34.9	98	62.4	97.3	0.001^a^
FAM	138	65.1	59	37.6	102.7	

^a^Chi-square test, ^b^Kruskal Wallis test, *p-value < 0.05 show significant association

## Discussion

This study demonstrated that most of the respondents aged 20 years old and below possessed good knowledge regarding HPV vaccination (56.4%). This had been agreed by Al-Naggar et al. (2010) with a study conducted among the general population showed that the majority of the respondents aged 17 to 30 had a good level of knowledge (82.4%) regarding HPV vaccine [7]. The younger population possessing good knowledge on HPV vaccination is parallel with the government’s effort since 2010 where the National HPV Immunisation Programme was launched for secondary schoolgirls. In addition, the NGOs have been actively involved in promoting HPV vaccination targeting the youth population.

The majority of the female respondents had a significantly good knowledge towards HPV vaccination (69.8%) as compared to the male respondents in this study. Similar findings were revealed in a study by O’Flarity (2012) where 83.0% of female respondents knew more about the HPV vaccine [8]. According to Ji et al. (2007), there was an association found between gender and knowledge towards HPV vaccine [9]. The different result between this study and the previous study is due to the difference in the distribution of respondents by ethnicity where in this study was not normally distributed as the majority of the respondents were Chinese.

This study found that there was a significant association between knowledge towards HPV vaccination at the *p*-value of 0.001 (*p* < 0.05). Similar findings were reported in the other studies [10, 11]. In addition, there was a significant association between parental income and level of knowledge on HPV vaccine. Respondents with low parental income showed good knowledge on HPV vaccination (52.5%) while parents with high income showed a poor knowledge regarding HPV vaccine (47.5%). In contrast, a similar study done among Malaysian women [12] found that respondents with a high family income had a good knowledge towards HPV vaccine.

Besides that, this study showed a significant association between age and attitude towards HPV vaccination as the *p*-value was 0.049 (*p* < 0.05). Respondents at age of 20 years old and below showed a positive attitude towards HPV vaccination as compared to the respondents aged beyond 20 (61.8% vs. 38.2%). A study among Malaysian women [12] also reported that there was significant association between attitude towards HPV vaccination and age. Respondents of the lower age range (19–30 years old) showed the highest percentage of positive attitude towards HPV vaccine (65.6%) while the higher age range (> 40 years old) had a higher percentage of negative attitude towards HPV vaccine (68.0%) [12]. Therefore, the lower the age, the higher the attitude towards HPV vaccine. According to gender difference, the female students showed a positive attitude towards HPV vaccination as compared to males. More than half of the respondents with positive attitudes were females (75.2%). A study among Korean adults [13] also found that adult females had a more positive attitude than adult males. This is due to less information about HPV vaccine related to male diseases. Most of the male respondents thought that HPV vaccine was just for women and related to cervical cancer.

In this study, the majority of the respondents with positive attitude towards HPV vaccine were respondents whose parents’ incomes were less than RM 3000, which was 59.9%. Another study showed that a family income of less than RM2000 had a more positive attitude (66.7%) when compared to a family income of more than RM 4000 (56.3%) [13]. Respondents with a lower socioeconomic status were more acceptable towards the HPV vaccine. In addition, this study reported that respondents with good knowledge were higher from FMHS (67.3%) when compared to FAM (32.7%). Another study also reported that a medical education had a direct correlation with a high level of knowledge where 87.7% of them had heard about HPV [14]. This is because medical students should know more about HPV vaccine when compared to students of other programmes since medical students will have more chance to get to know HPV vaccine during their classes and lectures. Most of the students with positive attitudes were from FMHS (62.4%) when compared to FAM (37.6%). This may be because the students from FMHS should know more and have a higher level of knowledge towards HPV vaccine. When these students understand the benefit of HPV vaccine and the consequence of HPV, they should have a more positive attitude towards HPV vaccine.

### Strengths and limitations

This study has contributed valuable facts and figures regarding HPV in relation to knowledge and attitude demonstrated among the students. However, the limitations of this study included the utilisation of Universiti Tunku Abdul Rahman (UTAR) as a private university and an imbalance in the demonstrated distribution of ethnicity and religion of the sample population. Besides that, due to the time duration of research, there is no assessment of vaccination status or intention could be done.

## Conclusion

The level of knowledge of UTAR students was reasonably high. Gender and parents’ income were associated with the knowledge of HPV vaccine while age and gender were associated with attitudes towards HPV vaccine. Nonetheless, a minority of male students still exhibited negative attitude towards HPV vaccination, depicting the necessity to impart and further intensify the sense of health awareness among all students. The judicious use of social media apart from the conventional mass media should be an advantage as to enhance the practice of HPV vaccination among them and thereafter minimize the health and economic burden of cervical cancer.

## Data Availability

Maiza Tusimin (MT) on behalf of all authors affirmed that the manuscript is an honest, accurate and transparent account of the study being reported. No important aspects of the study have been omitted and any discrepancies from the study as planned have been explained. The full dataset is available from the corresponding author maiza@upm.edu.my.

## Abbreviations

FAM: Faculty of Accountancy and Management
FMHS: Faculty of Medicine and Health Science
HPV: Human papillomavirus
NGOs: Non-government organisation
UTAR: Universiti Tunku Abdul Rahman
